# Peptide Frameworks as Microcosms of Metalloproteins

**DOI:** 10.1063/4.0000979

**Published:** 2025-10-27

**Authors:** Andy I Nguyen

**Affiliations:** University of Illinois Chicago, Chicago, IL, USA

## Abstract

Metalloenzymes efficiently catalyze some of the most challenging chemical transformations, many of which are critical to a sustainable society. Learning how to construct similar synthetic mimics by a bottom-up approach would both enhance our understanding of metalloenzymes and allow for the design of highly active artificial catalysts. Model compounds have long focused on the metal center(s), but it is now clear that the contribution of the protein scaffold needs to be addressed. However, the synthesis and structural characterization of elaborate protein-like ligands are major barriers towards the rational synthetic mimicry of the protein scaffold. To address these gaps, our laboratory has developed an approach to rapidly generate molecularly-defined, protein-like platforms via the self-assembly of peptides. We design small, chemically- synthesized peptides that assemble into porous crystalline materials (also called “frameworks”) that can bind metals and have multiple variable positions for rapid engineering of the secondary sphere. Furthermore, nearly all peptide frameworks form single crystals suitable for X-ray diffraction, allowing determination of detailed structural-functional relationships. The modularity and ease of peptide synthesis enables a practical synthetic approach to test how minimalist protein-like environments may elicit remarkable inorganic reactivity.

